# Brain imaging evidence for why we are numbed by numbers

**DOI:** 10.1038/s41598-020-66234-z

**Published:** 2020-06-09

**Authors:** Zheng Ye, Marcus Heldmann, Paul Slovic, Thomas F. Münte

**Affiliations:** 1 Institute of Neuroscience, Key Laboratory of Primate Neurobiology, CAS Center for Excellence in Brain Science and Intelligence Technology, Chinese Academy of Sciences, Shanghai, 200031 China; 2 Department of Neurology, University of Lübeck, 23538 Lübeck, Germany; 3 Institute of Psychologie II, University of Lübeck, 23538 Lübeck, Germany; 4 Decision Research, Eugene, Oregon 97401 USA; 5 Department of Psychology, University of Oregon, Eugene, Oregon 97403 USA

**Keywords:** Psychology, Human behaviour

## Abstract

We as humans do not value lives consistently. While we are willing to act for one victim, we often become numb as the number of victims increases. The empathic ability to adopt others’ perspectives is essential for motivating help. However, the perspective-taking ability in our brains seems limited. Using functional MRI, we demonstrated that the core empathy network including the medial prefrontal cortex (mPFC) was more engaged for events happening to a single person than those happening to many people, no matter whether the events were emotionally neutral or negative. In particular, the perspective-taking-related mPFC showed greater and more extended activations for events about one person than those about many people. The mPFC may be the neural marker of why we feel indifferent to the suffering of large numbers of people in humanitarian disasters.

## Introduction

On September 2, 2015, pictures of dead 3-year-old Alan Kurdi, a Kurdish boy from Syria, dominated the news globally, after he had drowned trying to reach the Greek island of Kos from Turkey with his family during the Middle East/European refugee crisis. Repercussions of the photo were dramatic. The British Prime Minister David Cameron said: “Anyone who saw those pictures overnight could not help but be moved and, as a father, I felt deeply moved by the sight of that young boy on a beach in Turkey.”^1^. On the days after the publication of the pictures, Google searches for the terms “Aylan”, “refugees”, and “Syria” increased sharply and daily donation amounts to the Swedish Red Cross increased 55-fold for a fund specifically designed to aid Syrian refugees^2^. The death of Alan Kurdi even heavily influenced the general elections of Canada^3^, the ultimate destination of Kurdi’s family. After that, the drowning of 700 refugees on May 26, 2016^4^, as well as other events involving greater numbers of casualties, has had considerably less media coverage and has not elicited compassionate responses by politicians.

The term compassion fade (or ‘collapse of compassion’) has been coined for this disturbing phenomenon: While we are willing to act and change policies in the aftermath of one identifiable victim, we are often passive and impervious to the fate of hundreds, thousands or even millions in large-scale crises^5–8^. This phenomenon is characterized by two tendencies in human altruistic behaviour^9–12^: the tendency to help a single victim more than multiple victims (the singularity effect), and the tendency to help a single identifiable victim more than an unidentifiable victim (the identifiability effect). The singularity effect and the identifiability effect are often observed in but may not be limited to emotionally negative scenarios (e.g., life-threatening disease, see Supplementary Information for further discussion). Even in emotionally neutral scenarios (e.g., giving professional advice), people tend to cheat more and give more biased advice to a group of people than a single person^13,14^. They also tend to treat an unidentifiable person less fairly than an identifiable person^14–16^. A previous study has provided brain imaging evidence for the identifiability effect^17^. Here we focus on the singularity effect.

Our willingness to act for other people seems diminished by numbers in both emotionally negative and neutral scenarios. But it is unclear as to why this is the case. One possibility is that human empathy does exactly what the colloquial description suggests: to put oneself in the place of one other person to fully understand his or her feelings. The empathic ability to adopt others’ perspectives is an essential component of compassionate responses (for a discussion of the differences between empathy and compassion, see^18–20^). The singularity effect may be mediated by empathic feelings^21^. People appeared to feel the most intense empathic emotions towards a specific person but these feelings are found to decrease as the number of needy people increases^10^. The decline of aroused empathic feelings and willingness to contribute has been observed as soon as the second victim is involved^22^.

Empathy is a multidimensional construct. Recent conceptualizations of human empathy have distinguished between a cognitive component, which involves intellectually understanding others’ intentions and behavioural outcomes, and an affective component, which involves vicariously experiencing others’ emotional and sensory states^18,23^. Cognitive empathy is often dubbed Theory of Mind. Social neuroscience research has identified a core network of human empathy, comprised of the medial prefrontal cortex (mPFC), middle cingulate cortex, and bilateral anterior insula^24^. In particular, the mPFC and middle cingulate cortex have been linked with cognitive empathy and the right anterior insula with affective empathy^25,26^. Furthermore, the mPFC has been selectively associated with the cognitive processes of perspective-taking^27–29^.

In this study, we focus on the empathic component of compassion fade. The singularity effect is more likely mediated by perspective-taking (cognitive empathy) rather than emotion sharing (affective empathy) because this effect exists in both emotionally negative and neutral scenarios. We hypothesize that the core network of human empathy, especially the key node mPFC, may be limited in its capacity to adopt others’ perspectives. In other words, the mPFC may tend to mentalize with and understand one person than a group of people. To test this hypothesis, we exposed 21 healthy young participants (10 women, age range 21-27 years, mean age 24.8 years) lying in a magnetic resonance imaging (MRI) scanner to a total of 20 new stories. The stories were composed in the format of a radio newscast and read by a trained actor in a neutral authoritative voice. Participants were asked to listen to the stories for comprehension. The stories either described rather neutral (e.g., a museum guide copying artwork) or intensely emotionally negative events (e.g., a rape; the Emotion factor) which either happened to a single person or many people (the Number factor). Given the absence of real helping behaviour (e.g., donation) in this task, we used a generalized definition of the singularity effect: the tendency to adopt the perspective of a single person more than many people (the general singularity effect). We expect to observe the general singularity effect regardless of the emotional valence of the story.

## Results

### Involvement of the mPFC

**Figure 1:**
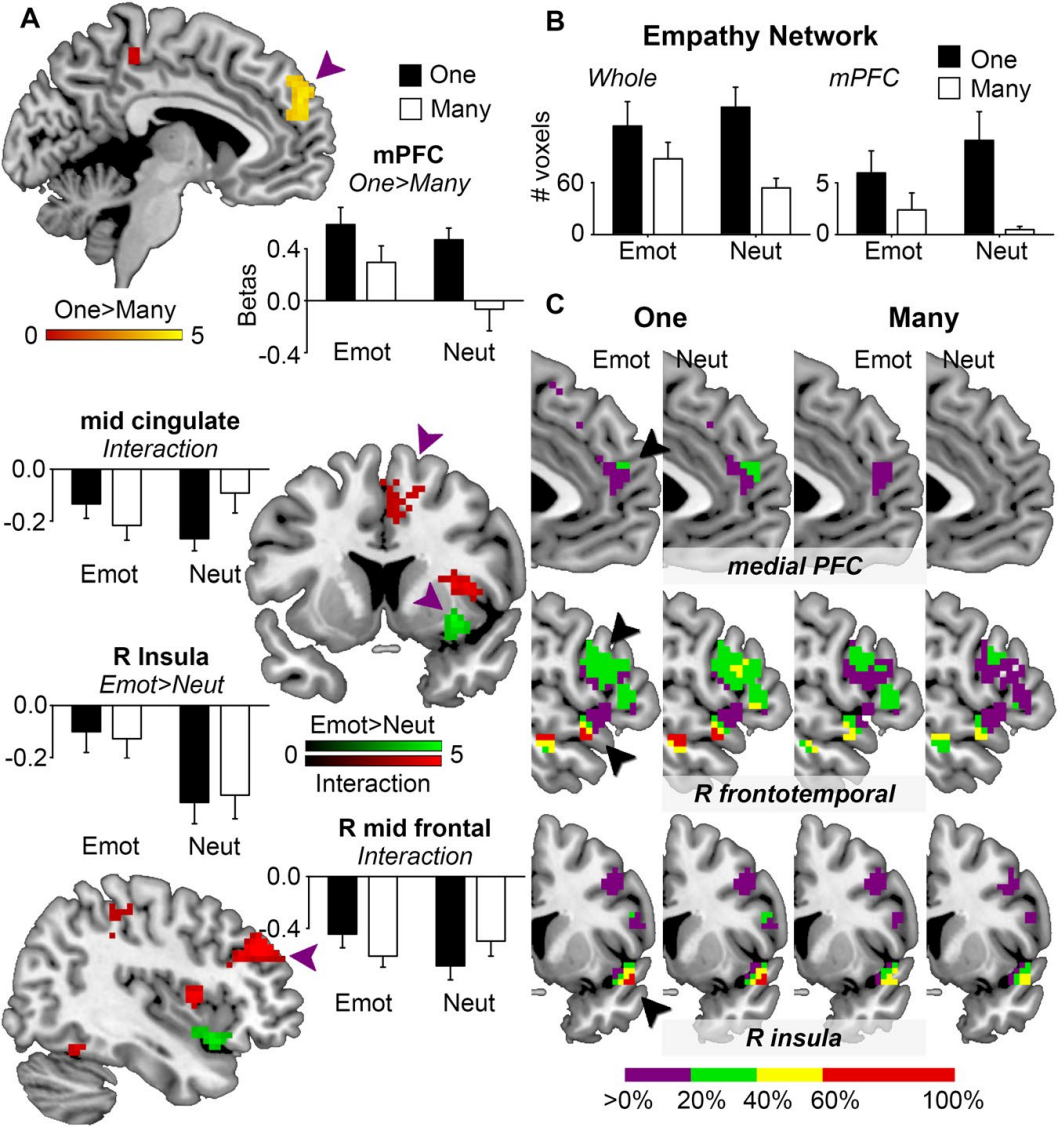
The core empathy network was more engaged for stories about one person than for stories about many people (the general singularity effect). (**A**) The medial prefrontal cortex (mPFC) showed greater activation for stories about one person than for stories about many people (One > Many, yellow-red scale). The right anterior insula showed less deactivation for emotionally negative than neural stories (Emot > Neut, green scale). The middle cingulate cortex and middle frontal cortex showed the general singularity effect, but only for emotionally negative stories (interaction, red scale). Colour scales indicate *t* values. Bar graphs present the mean estimated parameters of the general linear model (betas) for each story type for corresponding regions (purple arrows). Error bars indicate standard errors. R, right. (**B**) Bar graphs present the mean numbers of voxels that were significantly activated for each story type within the whole empathy network (left) and within the empathy-related mPFC (right). Error bars indicate standard errors. (**C**) Brain slices illustrate the overlap of individual participants’ activation maps with the core empathy network for each story type, zoomed in over the mPFC, right frontotemporal regions, and right insula. Colorbar indicates the percentage of overlapped participants. All imaging data were visualized with MATLAB (version r2015b, www.mathworks.com) and MRIcron (version 6.6.2013, www.mricro.com).

We first examined whether the mPFC or other empathy-related brain regions showed the general singularity effect, using a general linear model. In this model, we specified four story types by crossing the Emotion and Number factors, and a comprehensive index of head motion as a nuisance regressor to minimize potential effects of head motion^30^. Model parameters (beta weights) were estimated using classical parameter estimation. We found that the mPFC (peak coordinates in MNI space [-6 54 30], *t* = 4.78, cluster size 90 voxels) was more strongly activated for stories about one person than stories about many people, regardless of the emotional valence of the story (Fig. 1A, One > Many in the yellow-red scale, voxel-level *P* < 0.001 and cluster-level *P* < 0.05 family-wise-error corrected). In contrast, the right anterior insula ([33 9 -6], *t* = 5.48, 60 voxels) was sensitive to Emotion but not Number, showing less deactivation for emotionally negative stories than neutral stories (Fig. 1A, Emot > Neut in the green scale). We also observed an interaction between Number and Emotion over other brain regions, including the middle cingulate cortex ([12 -3 36], *t* = 5.11, 102 voxels), right middle frontal cortex ([36 42 33], *t* = 7.25, 123 voxels), right posterior insula ([36 6 9], *t* = 5.06, 66 voxels), right inferior temporal cortex ([57 -63 0], *t* = 4.89, 71 voxels), and right postcentral cortex ([45 -27 54], *t* = 4.51, 89 voxels). These brain regions were less deactivated for stories about one person than stories about many people, only when the stories expressed emotionally negative events (Fig. 1A, interaction in the red scale). No other regions were significant for the contrasts of interest.

### Contribution of the core empathy network

Having confirmed the involvement of the mPFC and other empathy-related brain regions, we then examined to what extent the core network of human empathy contributed to the general singularity effect. The core empathy network was derived from a meta-analysis of 112 functional imaging studies on empathic processes^24^. We obtained a mask image of the core empathy network from the ANIMA database^31^ and resampled the mask image to match the spatial resolution of current imaging data. We calculated the number of masked voxels that were significantly activated for each story type at the participant level (voxel-level *P* < 0.001 and cluster-level *P* < 0.05 family-wise-error corrected) and applied a Number-by-Emotion ANOVA (*P* < 0.025 for Bonferroni correction). Within the core empathy network, more voxels were significantly activated for stories about one person than stories about many people, regardless of the emotional valence of the story (F(1,20) = 15.50, *P* < 0.001, effect size partial *η*^2^ = 0.44; Fig. 1B left). We zoomed into the mPFC of the core empathy network and observed essentially a similar impact of Number (F(1,20) = 11.61, *P* < 0.005, partial *η*^2^ = 0.37; Fig. 1B right). Figure 1C illustrates the overlap of individual participants’ activation maps within the core empathy network for each story type.

### Contribution of the perspective-taking mPFC

**Figure 2:**
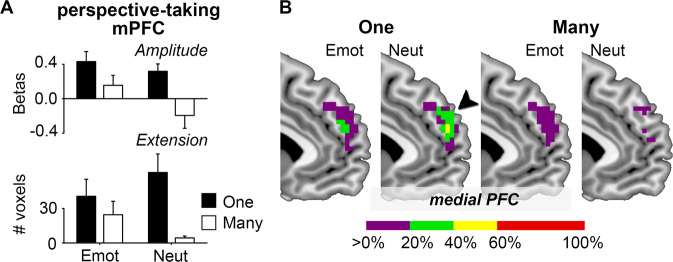
The regional activation of the perspective-taking mPFC was stronger for stories about one person than for stories about many people in terms of activation amplitude and spatial extension. (**A**) Bar graphs present the mean estimated model parameters (top) and the mean numbers of significantly activated voxels (bottom) in the perspective-taking mPFC for each story type. Error bars indicate standard errors. (**B**) Brain slices illustrate the overlap of individual participants’ activation maps with the perspective-taking mPFC. Colorbar indicates the percentage of overlapped participants. All imaging data were visualized with MATLAB (version r2015b, www.mathworks.com) and MRIcron (version 6.6.2013, www.mricro.com).

Finally, we tested the hypothesis that the perspective-taking-specific portion of the mPFC plays a role in the general singularity effect. For example, the mPFC may contribute by facilitating the understanding of others’ situations and needs. We expected that the regional activation of the perspective-taking mPFC was stronger for stories about one person than stories about many people in terms of activation amplitude and spatial extension. We obtained a mask image of the perspective-taking mPFC from a meta-analysis of 68 functional imaging studies related to perspective-taking^27^ and resampled the mask image to match the spatial resolution of current imaging data. Note, only 5% of the perspective-taking mPFC (20/395 voxels) overlapped with the mPFC of the core empathy network. Within this mask image, we calculated the estimated model parameters (amplitude) and the number of significantly activated voxels (spatial extension) at the participant level (voxel-level *P* < 0.001 and cluster-level *P* < 0.05 family-wise-error corrected) and applied two Number-by-Emotion ANOVAs (*P* < 0.025 for Bonferroni correction). We found that the perspective-taking mPFC showed greater activation (F(1,20) = 16.36, *P* < 0.001, partial *η*^2^ = 0.45; Fig. 2A top) and more activated voxels (F(1,20) = 11.30, *P* < 0.005, partial *η*^2^ = 0.36, Fig. 2A bottom) for one-person stories than many-people stories, regardless of the emotional valence of the story. Figure 2B illustrates the overlap of individual participants’ activation maps within the perspective-taking mPFC for each story type.

## Discussion

In an interview, Tima Kurdi, the aunt of Alan Kurdi said: “Don’t concentrate only on my family. Why are thousands of other Syrians in Turkey also so desperate?”^3^ But our brains are sensitive to an identifiable victim or an individualized entity like Kurdi’s family^22,32^. In this study, we showed that in healthy adults the core empathy network and its key node, mPFC, are the neural markers of why we feel indifferent to the suffering of large numbers of people. The core empathy network including the mPFC is seen to be much more engaged when we encounter stories about one person than stories about many people, no matter whether the stories are emotionally neutral or negative. In particular, the mPFC portion that has been selectively linked with perspective-taking showed greater and more extended activations for stories about one person than stories about many people. Our mPFC appears to be limited in its capacity to adopt others’ perspectives as the number of needy people increases.

The empathic ability to mentalize with and understand others is an essential component of compassion that eventually leads to altruistic behaviours^33^. But the limited perspective-taking capacity in the mPFC may potentially hamper our responses towards large-scale humanitarian problems such as refugee crises or genocides. From an egalitarian moral perspective, every human life should intrinsically be valued equally and the effort to allocate resources to help should increase proportionally as the number of victims increases. In the real world, however, protection of increasing numbers of human lives may not only have a decreasing marginal value^34^ but also have a decreasing absolute value at some point in both gain and loss frames^22,35^. An open question for future research is whether and how the subjective values of individual *versus* collective lives represented in the mPFC.

Observing that the tendency to mentalize with one person more than many people is built into our brains does not mean we should accept it as an excuse for acting passively when facing large-scale crises. This observation implies, however, that we can no longer rely on our moral intuitions. A potential solution might be to override this tendency by using “slow thinking” as proposed by dual-process theories of cognition^36^. This might help to create effective institutional mechanisms to deal with large-scale crises. However, finding a viable strategy for educating our imperfect empathy would not be easy. Simply teaching people about the inconsistent reactions evoked by one victim *versus* many victims may not enhance their willingness to contribute to help the many^37^. People still tend to rely on subjective preference even when they are aware of more effective options when helping others^38^. We leave this challenging issue for future research.

## Methods

All procedures were cleared by the ethical review board of the University of Magdeburg, the affiliation of the senior author at the time of the experiment, in accordance with the Declaration of Helsinki. All experiments were performed in accordance with relevant guidelines and regulations.

### Participants

Twenty-one native German speakers (10 women, mean age 24.7 years, age range 21-27 years) participated in this study. They were right-handed and had normal hearing. None of them had a history of neurological or psychiatric disorders. All of them gave written informed consent before participating in this study.

### Stimuli and task

The study used a slow event-related design (20 sec per trial with variable inter-trial intervals) to optimize fMRI. Participants listened to a total of 20 new stories via an MRI compatible headphone for comprehension. The stories were expressed in the format of a radio newscast and read by a trained actor in a neutral authoritative voice. The stories either described rather neutral or intensely emotionally negative events (the Emotion factor) which either happened to a single person or many people (the Number factor). Half of the many-people stories referred to a specific number (range between 24 and 50000), the other half used expressions such as “thousands of” or “numerous” (see Supplementary Information for additional comparisons between different types of many-people stories). The stories were between 75 and 83 words in length and had a duration of 20 sec. Stories were recorded in WAV-format (mono mode, 44.1 kHz sampling rate, sampling depth 16 bit).

### fMRI data acquisition

We acquired MRI data using a neuro-optimized 1.5-T GE Signa Horizon LX scanner with a standard quadrature head coil. Functional images were acquired using a standard T2*-weighted echo-planar imaging sequence (32 ascending interleaved axial slices, 2000-ms repetition time, 30-ms echo time, 80° flip angle, 224 × 224-mm^2^ field of view, 3.5-mm thickness, 1-mm gap, and 3.5 × 3.5-mm^2^ in-plane resolution). There were two experimental blocks, 346 volumes per block.

### fMRI data preprocessing

fMRI data were preprocessed and analyzed with SPM12 (www.fil.ion.ucl.ac.uk/spm). The first five volumes of each experimental block were discarded to allow magnetization equilibration. All functional images were realigned to the mean functional image, corrected for slice acquisition time difference using the middle slice as the reference slice, normalized to the MNI space using a segmentation function that segments, bias-corrects and spatially normalizes images in the same model^39^, smoothed with a Gaussian kernel of 8-mm full-width half-maximum, and filtered with a 128-sec high-pass filter.

We controlled the quality of the functional images using six motion parameters estimated in the realignment. First, no participant showed excessive head motion (translation >3 mm, or rotation >3 degrees). Second, we combined the six motion parameters into a more comprehensive index of head motion (total displacement) using the equation of Wilke^30^ and included the index as a nuisance regressor in the general linear model (GLM) to minimize potential effects of head motion (see below).

### Whole-brain univariate analysis

We first applied a whole-brain analysis to detect brain regions that were differentially activated in the processing of stories about one person *versus* stories about many people (One > Many, the general singularity effect). For each participant, the GLM convolved a design matrix with the canonical hemodynamic response function. The design matrix included four story types: emotional stories about one person (EmotOne), emotional stories about many people (EmotMany), neutral stories about one person (NeutOne), and neutral stories about many people (NeutMany). Each story was time-locked to its onset and modelled as a mini-block of 20 sec. Classical parameter estimation was applied with a one-lag autoregressive model. At the participant level, we defined the general singularity effect using the contrast of (EmotOne + NeutOne)>(EmotMany+NeutMany). At the group level, we used a one-sample *t*-test and considered results at voxel-level *P* < 0.001 and cluster-level *P* < 0.05 family-wise-error corrected to control false positives. Using similar approaches and thresholds, we also examined the main effect of Emotion (i.e. (EmotOne + EmotMany)>(NeutOne+NeutMany)), and the interaction between Number and Emotion (i.e. EmotOne-EmotMany)>(NeutOne-NeutMany)).

For visualization, we extracted estimated model parameters (beta weights) from each participant’s GLM from the medial prefrontal cortex (mPFC), middle cingulate cortex, right insula and right middle frontal cortex.

### Analysis of the core empathy network

We then examined to what extent the core empathy network contributed to the general singularity effect. The core empathy network was derived from a meta-analysis of 112 functional imaging studies on human empathy processes^24^. We obtained a mask image of the core empathy network from the ANIMA database^31^ and resampled the mask image to match the spatial resolution of current imaging data. We overlapped the mask image with each participant’s activation map (thresholded at voxel-level *P* < 0.001 and cluster-level *P* < 0.05 family-wise-error corrected) and calculated the number of overlapped voxels. For each participant, we calculated the numbers of overlapped voxels separately for each story type and entered them into an ANOVA with two factors, Number (One *versus* Many) and Emotion (Emot *versus* Neut). We considered the results at *P* < 0.025 (Bonferroni correction). We also applied the analysis for the mPFC of the core empathy network, using the same approaches and thresholds.

### Analysis of the perspective-taking mPFC

Finally, we examined whether the perspective-taking-specific portion of the mPFC showed the general singularity effect. The perspective-taking mPFC was derived from a meta-analysis of 68 functional imaging studies related to perspective-taking processes^27^. We obtained a mask image of the perspective-taking mPFC from the ANIMA database^31^ and resampled the mask image to match the spatial resolution of current imaging data. We then performed the analysis with two parameters: (a) the estimated model parameters (beta weights) extracted from each participant’s GLM for each story type, and (b) the number of overlapped voxels between the mask image and each participant’s activation map (thresholded at voxel-level *P* < 0.001 and cluster-level *P* < 0.05 family-wise-error corrected). We entered the parameters into two ANOVAs with Number and Emotion factors. We considered the results at *P* < 0.025 (Bonferroni correction).

## Supplementary information

Supplementary information.

## Data Availability

The data and the audio recordings are available at Dryad: doi:10.5061/dryad.wwpzgmsgj.
