# Supplementary material for: Brain imaging evidence for why we are numbed by numbers
Source: Sci Rep. 2020 Jun 9;10:9270. doi: 10.1038/s41598-020-66234-z (PMC7283346; doi:10.1038/s41598-020-66234-z)
Supplement: Supplementary file 1 — Supplementary information. [file 41598_2020_66234_MOESM1_ESM.docx]

Brain imaging evidence for why we are numbed by numbers

Zheng Ye^1,2^*, Marcus Heldmann^2,3^, Paul Slovic^4,5^, Thomas F. Münte^2,3^*

^1^ Institute of Neuroscience, Key Laboratory of Primate Neurobiology, CAS Center for Excellence in Brain Science and Intelligence Technology, Chinese Academy of Sciences, Shanghai 200031, China

^2^ Department of Neurology, University of Lübeck, 23538 Lübeck, Germany

^3^ Institute of Psychologie II, University of Lübeck, 23538 Lübeck, Germany

^4^ Decision Research, Eugene, Oregon 97401, USA

^5^ Department of Psychology, University of Oregon, Eugene, Oregon 97403, USA

*Correspondence: thomas.muente@neuro.uni-luebeck.de (TFM), yez@ion.ac.cn (ZY)

**Supplementary Information**

*Effects of singularity and identifiability in neutral scenarios*

The effects of singularity and identifiability are often observed in but may not be limited to emotionally negative scenarios. We reviewed scenarios and contrasts of all studies included in a recent meta-analysis of compassion fade ^1^. Most of the studies only used emotionally negative scenarios (e.g., life-threatening disease, starvation, and war), without a direct comparison between neutral and negative scenarios (Table S1). This is not surprising because the term ‘compassion fade’ emphasizes the lack of willingness to help in the face of large-scale crises which are intensely emotionally negative. Note, we excluded some previous studies and explained the reasons in Table S2.

Among these studies, six studies included neutral scenarios (e.g., playing a dictator game) and four of them reported the effect of singularity or identifiability (Table S3). In brief, people tend to cheat more and give more biased advice to a group of people than a single person ^2,3^. They also tend to allocate money more fairly and give less biased advice to a single identifiable than an unidentifiable person ^3-5^. Our willingness to help seems diminished by numbers regardless of emotional valence. Therefore, we hypothesize that people tend to mentalize with a single person more than a group of people even in neutral scenarios.

Table S1: Emotionally negative scenarios used in previous studies

| **Original articles** | **Description of scenarios and contrasts** |
| --- | --- |
| *Helping children in danger from a life-threatening disease* | |
| Ambrona, et al. ^6^ | Study 1&2: a single identifiable child vs. the same child as one among many  Study 3: a single identifiable child vs. the same child as one among many vs. statistical data |
| Dickert and Slovic ^7^ | Study 1&2: a single identifiable child vs. the same child as one among many |
| Dickert, et al. ^8^ | Study 1: 1 vs. 8 identifiable children |
| Han ^9^ | Study 1: a single identifiable child vs. statistical data |
| Jenni and Loewenstein ^10^ | Study 2: a single identifiable child vs. statistical data |
| Kogut and Ritov ^11^ | Study 1&2: 1 vs. 8 identifiable/unidentifiable children  Study 3: 1 vs. 8 identifiable children |
| Kogut and Ritov ^12^ | Study 1: 1 vs. 8 identifiable/unidentifiable children  Study 2: 1 vs. 7 identifiable children  Study 3: 1 vs. 7 identifiable children, in need or not |
| Kogut and Ritov ^5^ | Study 2: 1 vs. 7 identifiable children, in- or out-group |
| Kogut, et al. ^13^ | Study 1-3: 1 vs. 8 identifiable children |
| Wang, et al. ^14^ | 1 vs. 8 identifiable/unidentifiable children |
| *Helping children in danger of starvation and malnutrition* | |
| Dickert, et al. ^15^ | Study 2: 1 vs. 5 identifiable/unidentifiable children |
| Dickert, et al. ^16^ | Study 1&2: 1 vs. 5 identifiable/unidentifiable children |
| Friedrich and McGuire ^17^ | a single identifiable child vs. statistical data vs. both |
| Lee and Feeley ^18^ | Study 1&2: a single identifiable child vs. statistical data |
| Lesner and Rasmussen ^19^ | a single identifiable child vs. statistical data |
| Small, et al. ^20^ | a single identifiable victim vs. statistical data, with or without teaching/priming |
| Västfjäll, et al. ^21^ | 1 vs. 2 vs. 8 identifiable victims |
| Västfjäll, et al. ^22^ | a single determined vs. undetermined victim |
| *Helping the victims of war* | |
| Cameron and Payne ^23^ | Study 1&3: 1 vs. 8 identifiable children in Darfur  Study 2: 1 vs. 4 vs. 8 identifiable children in Darfur |
| Erlandsson, et al. ^24^ | Study 1&2: a single identifiable child vs. 9 unidentifiable children from Syria  Study 3: a single identifiable child vs. statistical data |
| Slovic, et al. ^25^ | Alan Kurdi vs. statistical data of the Syrian war |
| *Helping the poor/underprivileged* | |
| Cryder and Loewenstein ^26^ | Study 2: a single determined vs. undetermined needy girl in Colombia and Chile, with or without a picture |
| Ein-Gar and Levontin ^27^ | Study 1: a single identifiable underprivileged child vs. an association that helps such children |
| Erlandsson, et al. ^28^ | Study 1: a single identifiable child in Mozambique vs. a single unidentifiable child vs. statistical data |
| Kogut and Slovic ^29^ | a single identifiable child vs. 6 children from a different school |
| Lunt ^30^ | a single identifiable homeless young man vs. an association that helps homeless young men |
| Oceja, et al. ^31^ | Study 1: a single identifiable child vs. the same child as one among many |
| Oceja, et al. ^32^ | Study 1&2: a single identifiable child in Sierra Leone vs. the same child as one among many |
| Oceja and Salgado ^33^ | Study 2: a single identifiable child in Sierra Leone vs. the same child as one among many |
| Rubaltelli and Agnoli ^34^ | Study 1&2: a single identifiable woman vs. 3 women in Rwanda |
| Sudhir, et al. ^35^ | a single identifiable old woman vs. 4 unidentifiable old women |
| *Other scenarios* | |
| Kogut and Ritov ^5^ | Study 1: 1 vs. 7 identifiable/unidentifiable earthquake victims, in- or out-group |
| Kogut ^36^ | Study 1: a single identifiable young man with AIDS vs. a group of young men with AIDS  Study 4: a single identifiable single mother vs. an association that help single mothers |

Table S2: Excluded studies

| **Original articles** | **Reasons for exclusion** |
| --- | --- |
| Cryder, et al. ^37^ | using stories about a charity organization (e.g., Oxfam International) rather than human victims |
| Deshpande and Spears ^38^ | unpublished manuscript |
| Dickert ^39^ | unpublished doctoral dissertation |
| Kleber, et al. ^40^ | contrasting an absolute number with a proportion |
| Kogut, et al. ^41^ | unpublished manuscript |
| Kogut, et al. ^42^ | unpublished manuscript |
| Hsee and Rottenstreich ^43^ | contrasting an object/animal with multiple objects/animals |
| Kohn, et al. ^44^ | contrasting a gravely ill patient with a potential organ donor |
| Markowitz, et al. ^45^ | contrasting a single animal with multiple animals |
| Smith, et al. ^46^ | studying the effect of entitativity |
| Weiner ^47^ | studying the effect of responsibility |

Table S3: Effects of singularity or identifiability in neutral scenarios

| **Original articles** | **Scenarios and contrasts** | **Results** |
| --- | --- | --- |
| Amir, et al. ^2^ | cheating a single person vs. a group of people | Effect of singularity |
| Cryder and Loewenstein ^26^ | playing a dictator game with a single identifiable vs. an unidentifiable person | No effect |
| Kogut and Ritov ^12^ | helping a single identifiable vs. an unidentifiable gifted child | No effect |
| Ritov and Kogut ^48^ | playing a dictator game with a single identifiable vs. an unidentifiable person, in- or out-group | Effects of identifiability and group cohesiveness |
| Sah and Loewenstein ^3^ | giving biased advice to a single person vs. multiple persons and to a single identifiable vs. an unidentifiable person | Effects of singularity and identifiability |
| Small and Loewenstein ^4^ | playing a dictator game with a single determined vs. an undetermined person | Effects of determination (a weak form of identifiability) |

*The core empathy network did not track the number of people in many-people stories*

The number of people in many-people stories varied from “two dozens” to “thousands”. We conducted a separate general linear model to examine whether the core empathy network tracked the number of people. To this end, we categorized the many-people stories into stories about 24-50 people (‘Many1’, N=4), and stories about thousands of people (‘Many2’, N=6). At the subject level, the model included four types of stories: emotional one-person stories, neutral one-person stories, ‘Many1’ stories, and ‘Many2’ stories. We no longer separated the emotional and neutral many-people stories because the numbers of trials were limited.

First, we replicated the general singularity effect in the mPFC (One>Many1+2, whole-brain one-sample *t*-test, voxel-level *P* < 0.001 and cluster-level *P* < 0.05 family-wise-error corrected; coordinates in MNI space [-6 54 30], *t* = 4.87, cluster size 91 voxels). However, we observed no significant regional activation for Many1>Many2 stories (see Fig.S1).

Second, we extracted estimated model parameters (beta weights) from the mPFC (from the contrast One>Many1+2) and the core empathy network (from the ANIMA database) for a region-of-interest analysis (paired *t*-tests, *P* < 0.05). We observed no significant difference between Many1 and Many2 stories within the mPFC (*t* < 1) or core empathy network (*t*(20) = 1.41, *P* = 0.17; see Fig.S1).

In summary, both whole-brain analysis and region-of-interest analysis suggested that the core empathy network did not track the number of people in many-people stories.

Fig.S1: The medial prefrontal cortex (mPFC) showed greater activation for stories about one person than for all types of stories about many people (One>Many1+2). No regions showed greater activation for Many1 *versus* Many2 stories (Many1>Many2). Colour scale indicates t values. Line graphs present means and standard errors of the estimated model parameters (betas) for each story type for the mPFC and core empathy network. Imaging data were visualized with MATLAB (version r2015b, www.mathworks.com) and MRIcron (version 6.6.2013, www.mricro.com).


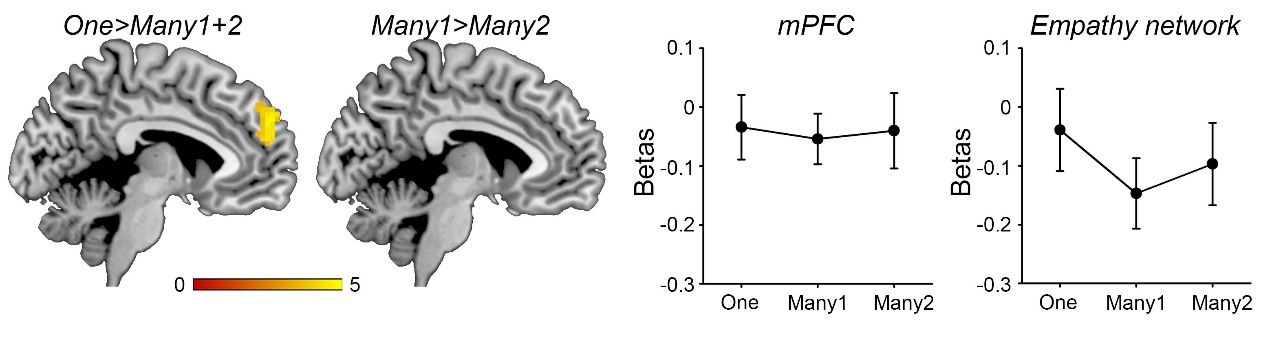


**References**

1 Butts, M. M., Lunt, D. C., Freling, T. L. & Gabriel, A. S. Helping one or helping many? A theoretical integration and meta-analytic review of the compassion fade literature. *Organ. Behav. Hum. Decis. Process.* **151**, 16-33 (2019).

2 Amir, A., Kogut, T. & Bereby-Meyer, Y. Careful cheating: People cheat groups rather than individuals. *Frontiers in Psychology* **7**, 371 (2016).

3 Sah, S. & Loewenstein, G. More affected = more neglected: Amplification of bias in advice to the unidentified and many. *Social Psychological and Personality Science* **3**, 365–372 (2012).

4 Small, D. A. & Loewenstein, G. Helping a victim or helping the victim: Altruism and identifiability *Journal of Risk and Uncertainty* **26**, 5–16 (2003).

5 Kogut, T. & Ritov, I. "One of us": Outstanding willingness to help rescue a single identified compatriot. *Organ. Behav. Hum. Decis. Process.* **104**, 150–157 (2007).

6 Ambrona, T., Oceja, L., Lopez-Perez, B. L. & Carrera, P. Can empatheic concern be generlaized from one person to others? Another positive side of the 'one-among-ohters' effect. *Scandinavian Journal of Psychology* **57**, 547-553 (2016).

7 Dickert, S. & Slovic, P. Attentional mechanism in the generation of sympathy. *Judg Dec Mak* **4**, 297–306 (2009).

8 Dickert, S., Sagara, N. & Slovic, P. Affective motivations to help others: A twostage model of donation decisions. *Journal of Behavioral Decision Making* **24**, 361–376 (2011).

9 Han, V. *The role of self-construal level on message evidence in cause-related*

*marketing advertising campaigns* master thesis, University of Texas, (2013).

10 Jenni, K. E. & Loewenstein, G. Explaining the “identifiable victim effect”. *Journal of Risk and Uncertainty* **14**, 235–257 (1997).

11 Kogut, T. & Ritov, I. The “identified victim” effect: An identified group, or just a single individual? *Journal of Behavioral Decision Making* **18**, 157–167 (2005).

12 Kogut, T. & Ritov, I. The singularity effect of identified victims in separate and joint evaluations. *Organ. Behav. Hum. Decis. Process.* **97**, 106-116 (2005).

13 Kogut, T., Slovic, P. & Västfjäll, D. Scope insensitivity in helping decisions: Is it a matter of culture and values? *Journal of Experimental Psychology: General* **144**, 1042–1052 (2015).

14 Wang, Y., Tang, Y.-T. & Wang, J. Cultural differences in donation decision-making. *PLoS ONE* **10**, e0138219 (2015).

15 Dickert, S., Kleber, J., Peters, E. & Slovic, P. Numeracy as a precursor to prosocial behavior: The impact of numeracy and presentation format on the cognitive mechanisms underlying donation decisions. *Judgement and Decision Making* **6**, 638–650 (2011).

16 Dickert, S., Kleber, J., Västfjäll, D. & Slovic, P. Mental imagery, impact, and affect: A mediation model for charitable giving. *PLoS One* **11**, e0148274 (2016).

17 Friedrich, J. & McGuire, A. Individual differences in reasoning style as a moderator of the identifiable victim effect. *Social Influence* **5**, 182–201 (2010).

18 Lee, S. & Feeley, T. H. The identifiable victim effect: Using an experimentalcausal-chain design to test for mediation. *Current Psychology* **37**, 875–885 (2018).

19 Lesner, T. H. & Rasmussen, O. D. The identifiable victim effect in charitable giving: evidence from a natural field experiment. *Applied Economics* **46**, 4409–4430 (2014).

20 Small, D. A., Loewenstein, G. & Slovic, P. Sympathy and callousness: The impact of deliberative thought on donations to identifiable and statistical victims. *Organ. Behav. Hum. Decis. Process.* **102**, 143-153 (2007).

21 Västfjäll, D., Slovic, P., Mayorga, M. & Peters, E. Compassion fade: Affect and charity are greatest for a single child in need. *PLoS ONE* **9**, e100115 (2014).

22 Västfjäll, D., Slovic, P. & Mayorga, M. Pseudoineffacy: Negative feelings from children who cannot be helped reduce warm glow for children who can be helped. *Frontiers in Psychology* **6**, 616 (2015).

23 Cameron, C. D. & Payne, B. K. Escaping affect: How motivated emotion regulation creates insensitivity to mass suffering. *Journal of Personality and Social Psychology* **100**, 1-15 (2011).

24 Erlandsson, A., Västfjäll, D., Sundfelt, O. & Slovic, P. Argument-inconsistency in charity appeals: Statistical information about the scope of the problem decrease helping toward a single identified victim but not helping toward many non-identified victims in a refugee crisis context. *Journal of Economic Psychology* **56**, 126–140 (2016).

25 Slovic, P., Västfjäll, D., Erlandsson, A. & Gregory, R. Iconic photographs and the ebb and flow of empathic response to humanitarian disasters. *Proc Natl Acad Sci U S A* **114**, 640-644 (2017).

26 Cryder, C. E. & Loewenstein, G. Responsibility: The tie that binds. *Journal of Experimental Social Psychology* **48**, 441-445 (2012).

27 Ein-Gar, D. & Levontin, L. Giving from a distance: Putting the charitable organization at the center of the donation appeal. *Journal of Consumer Psychology* **23**, 197–211 (2013).

28 Erlandsson, A., Björklund, F. & Bäckström, M. Emotional reactions, perceived impact and perceived responsibility mediate the identifiable victim effect, proportion dominance effect and in-group effect respectively. *Organ. Behav. Hum. Decis. Process.* **127**, 1–14 (2015).

29 Kogut, T. & Slovic, P. The development of scope insensitivity in sharing behavior *Journal of Experimental Psychology: Learning, Memory, and Cognition* **42**, 1972-1981 (2016).

30 Lunt, D. L. *Willing to help, but lacking discernment: The effects of victim group size on donation behaviors* Ph.D. thesis, University of Texas-Arlington, (2016).

31 Oceja, L., Ambrona, T., López-Pérez, B., Salgado, S. & Villegas, M. When the victim is one among others: Empathy, awareness of others and motivational ambivalence. *Motivation and Emotion* **34**, 110–119 (2010).

32 Oceja, L., Stocks, E. & Lishner, D. Congruence between the target in need and the recipient of aid: The one-among-others effect. *Journal of Applied Social Psychology* **40**, 2814–2828 (2010).

33 Oceja, L. & Salgado, S. Why do we help? World change orientation as an antecedent of prosocial action. *European Journal of Social Psychology* **43**, 127–136 (2013).

34 Rubaltelli, E. & Agnoli, S. The emotional cost of charitable donations. *Cognition and Emotion* **26**, 769–785 (2012).

35 Sudhir, K., Roy, S. & Cherian, M. Do sympathy biases induce charitable giving? The effects of advertising content. *Marketing Science* **35**, 849–869 (2016).

36 Kogut, T. Someone to blame: When identifying a victim decreases helping. *Journal of Experimental Social Psychology* **47**, 748–755 (2011).

37 Cryder, C. E., Loewenstein, G. & Scheines, R. The donor is in the details. *Organ. Behav. Hum. Decis. Process.* **120**, 15-23 (2013).

38 Deshpande, A. & Spears, D. *Who is the identifiable victim? Caste interacts with sympathy in India* (University of Delhi, Delhi, India, 2012).

39 Dickert, S. *Two routes to the perception of need: The role of affective and deliberative information processing in prosocial behavior* Ph.D. thesis, University of Oregon, (2008).

40 Kleber, J., Dickert, S., Peters, E. & Florack, A. Same numbers, different meanings: How numeracy influences the important of numbers for pro-social behavior. *Journal of Experimental Social Psychology* **49**, 699–705 (2013).

41 Kogut, T., Slovic, P. & Västfjäll, D. *The effect of the recipient’s identifiability and neediness on children’s sharing behavior* (Ben-Gurion University of the Negev, Beer-Sheva, Israel, 2014).

42 Kogut, T., Slovic, P. & Västfjäll, D. *Understanding the developmental sources of scope insensitivity in sharing behavior* (Ben-Gurion University of the Negev, Beer-Sheva, Israel, 2014).

43 Hsee, C. K. & Rottenstreich, Y. Music, pandas, and muggers: On the affective psychology of value. *Journal of Experimental Psychology: General* **133**, 23–30 (2004).

44 Kohn, R., Rubenfeld, G. D., Levy, M. M., Ubel, P. A. & Halpern, S. D. Rule of rescure or the good of the many? An analysis of physicians’ and nurses’ preferences for allocating ICU beds. *Intensive Care Medicine* **37**, 1210–1217 (2011).

45 Markowitz, E. M., Slovic, P., Västfjäll, D. & Hodges, S. D. Compassion fade and the challenge of environmental conservation. *Judg Dec Mak* **8**, 397–406 (2013).

46 Smith, R. W., Faro, D. & Burson, K. A. More for the many: The influence of entitativity on charitable giving. *Journal of Consumer Research* **39**, 961–976 (2013).

47 Weiner, B. A cognitive (attribution)-emotion-action model of motivated behavior: An analysis of judgments of help-giving. *Journal of Personality and Social Psychology* **39**, 186–200 (1980).

48 Ritov, I. & Kogut, T. Altruistic behavior in cohesive social groups: The role of target identifiability. *PLoS One* **12**, e0187903 (2017).
